# How I do it open distal ureteroureterostomy for ectopic ureters in infants with duplex systems and no vesicoureteral reflux under 6 months of age

**DOI:** 10.1590/S1677-5538.IBJU.2020.0742

**Published:** 2020-10-20

**Authors:** Yuding Wang, Luis H. Braga

**Affiliations:** 1 McMaster University Departament of Surgery Hamilton Canada Departament of Surgery, McMaster University, Hamilton, Canada

**Keywords:** Vesico-Ureteral Reflux, Ureter, Infant

## Abstract

We describe a step by step technique for open distal ureteroureterostomy (UU) in infants less than 6 months presenting with duplex collecting system and upper pole ectopic ureter in the absence of vesicoureteral reflux (VUR).

## BACKGROUND

Approximately 70% of ectopic ureters occur with ureteral duplication, with 80% of cases including contralateral duplication. They are found mainly in Caucasian children and are four to seven times more common in females (1). The typical patient used to present with recurrent UTIs, flank pain from obstruction, or continuous incontinence (2). However, currently, the great majority of these patients are discovered incidentally on routine investigation of prenatal hydronephrosis (2).

It has been common practice to defer surgical intervention of the patients diagnosed in the neonatal period until 12 months of age. Possible management options include upper pole heminephrectomy, pyeloureterostomy, common sheath ureteral reimplantation, upper pole ureteral clipping, and ipsilateral proximal or distal ureteroureterostomy. The decision towards an ablative versus a reconstructive approach has traditionally been influenced by upper pole function, as well as surgeon preference. Recent studies have demonstrated the feasibility, efficacy and minimally invasiveness of the open distal UU for management of patients with upper pole ectopic ureters without VUR (3–6). The potential risk of injury to the normal lower pole ureter from the dissection and anastomosis has not been described yet. Furthermore, recent evidence has challenged the adage that having a poorly functional upper pole moiety (defined at <10%) is an indication for ablative management (4). The upper pole heminephrectomy may in fact cause more harm by damaging the normal healthy tissue of lower pole moiety during the surgical resection, as previously reported (4).

Open distal UU can be performed safely at any age, but specially in the first 6 months of life. The thin abdominal wall and the shallow pelvis of the young infant allow for an easy and straightforward access to the distal ureters. In doing such an approach, the infant may receive definite management negating the risk of continued renal damage to the upper pole moiety from persistent obstruction, and/or the risk of UTIs or urosepsis requiring hospitalization. Open distal UU has been the technique of choice to manage duplex systems with upper pole ectopic ureters without VUR in our institution. Since 2008, we have performed 58 cases. The mean age at surgery was 6 months, mean operative time was 70 minutes and 95% of patients demonstrated resolution of their hydronephrosis. No reoperations have been noted thus far. Herein, we present the surgical steps of the open distal UU technique, highlighting tips and tricks that make this procedure effective and reproducible.

## SURGICAL PROCEDURE

**Figure 1:**
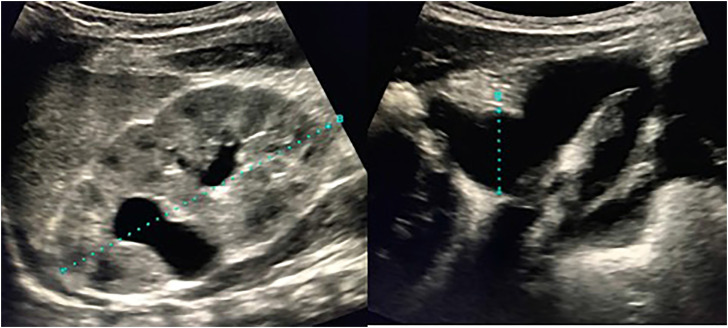
Preoperative ultrasound demonstrating severe hydroureter and hydronephrosis to the upper pole moiety of a duplex collecting system.

The infant is placed in the supine position and the incision site is marked at the inguinal crease, similar to an inguinal orchidopexy. Cystoscopy and retrograde pyelogram can be obviated, as patients have duplex systems with unilateral upper pole hydroureteronephrosis on ultrasound (Figure-1) and absence of VUR on voiding cystourethrogram, which is highly suggestive of ureteral ectopia. If one is not certain of the diagnosis, cystoscopy can be performed immediately before the procedure looking for the presence of a single ipsilateral ureteral orifice in the bladder, which confirms the ectopic location of the upper pole ureter somewhere below the bladder. Attempting to identify the exact insertion of the ectopic ureter only leads to delay in starting time, as the ectopic orifice is not always identifiable.

**Figure 2:**
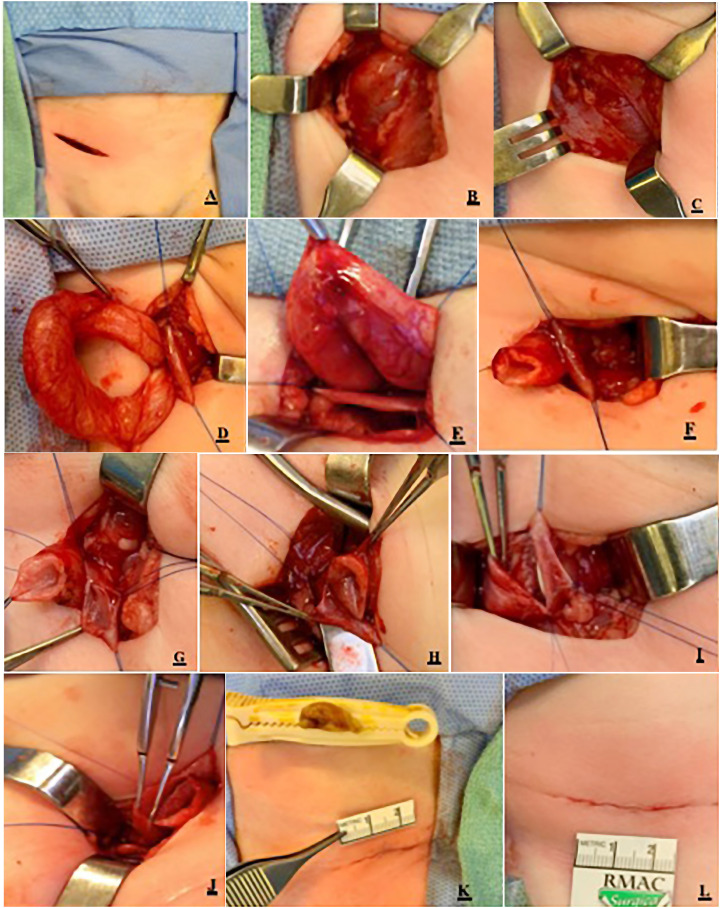
Step by step technique of distal ureteroureterostomy on a 3-week-old girl presenting with urosepsis due to an obstructing upper pole moiety of an ectopic ureter. A/B. Oblique incision made on the external oblique fascia. C. Muscle splitting technique is used until peritoneum is reached and retracted to allow access into the retroperitoneal space. D/E. Obstructing upper pole ureter is separated from normal lower pole ureter. F/G/H. Obstructed ureter is cut and spatulated, reducing its size considerably. I/J. End to side anastomosis is carried out using a running suture of 7-0 PDS. I. Stent is inserted under direct vision before completion of the anastomosis. K/L. Skin closure with subcuticular 4-0 Monocryl.

A small 2-3cm transverse inguinal incision is made along Langer's lines at the inguinal crease on the ipsilateral side (Figure-2a). Scarpa's fascia is dissected and the external oblique's fascia is incised obliquely to allow for better exposure (Figure-2b). Muscle splitting approach was utilized to reach the transverse's fascia which was opened to allow access into the retroperitoneal region (Figures-2b and c). A virtual space is created by pushing a wet gauze into that area to allow for creation of the working space without entering the peritoneum.

Once in the retroperitoneal space, dissection is completed slightly caudally and medially to identify three important anatomical landmarks: the obliterated umbilical artery, the iliac vessels and the psoas muscle. These three structures will help and lead to the identification of the dilated upper pole ureter, which sometimes can be mistaken by a bowel loop due to its substantial caliber. There is no need to look for the small caliber lower pole ureter, as it is always attached to the wall of the upper pole ureter and will be identified during the dissection of the “big ureter”. Trying to insert a 3Fr-feeding tube in the lower pole ureter during cystoscopy to facilitate the intraoperative identification of the lower pole ureter is not necessary and, sometimes, harmful, as it may cause injury (edema) of the lower pole ureteric orifice while attempting its endoscopic catheterization.

The ureters are dissected “en-bloc” superiorly and inferiorly (towards the bladder), and delivered through the incision to facilitate exposure (Figure-2d). A self-retaining (Weitlaner) retractor can be placed at this point allowing proper exposure, obviating the need to use divers or other retractors. The dilated upper pole ureter is separated from the normal lower pole ureter, and stay sutures with 5-0 Prolene are placed to set up the exposure for the anastomosis (Figure-2e). Care is taken not to over dissect the lower pole ureter for risk of vascular compromise. A good trick here is to leave the upper pole ureter wall attached to the lower pole to avoid devascularization of that ureter (Figures-2f-h). The upper pole ureter should be spatulated as distally as possible, in proximity to the bladder wall and left open, as there is no risk of urine leak because of the obstructive nature of the upper pole ectopic ureter in these cases. In rare cases of concurrent obstruction and VUR, then this upper pole ureter is ligated as low as possible to avoid leaving a long distal ureteric stump that could be a source for urinary tract infection in the future. Care should also be taken not to over dissect the lower pole ureter proximally to preserve its vascular supply.

Once dissection is finished, an end to side anastomosis is completed using running 7-0 PDS (Figures-2i-j). Care should be taken to ensure natural lay, absence of tension, and a water-tight mucosa to mucosa anastomosis. Tapering of the dilated upper pole ureter is not necessary to achieve a successful outcome (7). The trick here is to measure the diameter of the upper pole lumen and create a spatulation of the same size in the recipient lower pole ureter (Figures-2f and g). A stent is placed under vision though one is not mandatory (5). Once stenting is chosen, it can be placed in the lower pole ureter or trans-anastomotic into the upper pole ureter, with similar results (Figure-2i). Patency of the distal lower pole ureteral lumen is checked before completing the anterior part of the anastomosis, as clearly demonstrated in this (Figure-2j). The transverse and internal oblique muscles are approximated with a running suture of 4-0 vicryl and the external oblique fascia is closed with 4-0 PDS. Subcutilar skin closure is performed with 5-0 monocryl to achieve an excellent cosmetic result (Figures-2k and l).

A foley catheter is kept in place until patient is discharged, which can happen later on the same day or next day. When inserted, stents are removed in 3-4 weeks under general anesthesia.

## CONCLUSIONS

An open distal UU can be performed safely and effectively through a small incision in the inguinal crease in infants younger than 6 months of age, with minimal morbidity and short hospital stay. Its excellent cosmesis and minimally invasive nature are comparable to any robotic or laparoscopic procedures.
